# Vagus activation by Cold Face Test reduces acute psychosocial stress responses

**DOI:** 10.1038/s41598-022-23222-9

**Published:** 2022-11-10

**Authors:** Robert Richer, Janis Zenkner, Arne Küderle, Nicolas Rohleder, Bjoern M. Eskofier

**Affiliations:** 1 Machine Learning and Data Analytics Lab (MaD Lab), Department Artificial Intelligence in Biomedical Engineering (AIBE), Friedrich-Alexander-Universität Erlangen-Nürnberg (FAU), 91052 Erlangen, Germany; 2 Chair of Health Psychology, Friedrich-Alexander-Universität Erlangen-Nürnberg (FAU), 91052 Erlangen, Germany

**Keywords:** Human behaviour, Biomedical engineering

## Abstract

Chronic stress is linked to dysregulations of the two major stress pathways—the sympathetic nervous system and the hypothalamic-pituitary-adrenal (HPA) axis, which could for example result from maladaptive responses to repeated acute stress. Improving recovery from acute stress could therefore help to prevent this dysregulation. One possibility of physiologically interfering with an acute stress reaction might be provided by applying a cold stimulus to the face (Cold Face Test, CFT) which activates the parasympathetic nervous system (PNS), leading to immediate heart rate decreases. Therefore, we investigated the use of the CFT protocol as an intervention to reduce acute stress responses. Twenty-eight healthy participants were exposed to acute psychosocial stress via the Montreal Imaging Stress Task (MIST) in a randomized between-subjects design while heart rate (HR), heart rate variability (HRV), and salivary cortisol were assessed. While both groups were equally stressed during the procedure, participants with CFT intervention showed better recovery, indicated by significant ($p<0.05$) differences in HR(V). We additionally found a significantly ($p<0.05$) lower cortisol response to the MIST and less overall cortisol secretion in the CFT condition. Both findings indicate that the CFT can successfully stimulate the PNS and inhibit the HPA axis. To the best of our knowledge, our experiment is the first to successfully use the CFT as a simple and easy-to-apply method to modify biological responses to acute stress.

## Introduction

In 2017, the European Commission reported that “work-related stress is among the most challenging—and growing—occupational safety and health concerns”^1^. The economic impact of stress is huge: About half of all workplace absences can be linked to stress. The total cost of work-related depression in Europe alone was estimated to be €617 billion annually^2^.

However, despite the widespread opinion that stress is generally “unhealthy”, physiological stress responses are actually crucial to survival since they set the human body into a state of alertness, thereby allowing it to rapidly adapt to new situations or challenges. An acute stress response is based on the complex interplay between different pathways: It is initiated by stress centers in the central nervous system and communicated to the body by the activation of the hypothalamic-pituitary-adrenal (HPA) axis and the sympathetic nervous system (SNS). Increased HPA axis activity leads to the secretion of cortisol, one of the most important stress markers, while stimulation of the SNS induces the “fight or flight” response, inducing increases in adrenaline, heart rate, and blood pressure, and increased levels of arousal and alertness^3^. After the end of a stressful situation, negative feedback of the HPA axis and activation of the parasympathetic nervous system (PNS), thus initiating the “rest and digest” reaction, shut down the stress response. Measuring these physiological responses can be used to track stress-related changes. Most commonly, this is performed by the observation of changes in the human body’s electrophysiology, such as heart rate (HR) and heart rate variability (HRV), or electrodermal activity (EDA)^4^. Concurrently, neuroendocrine biomarkers, such as cortisol or alpha-amylase, measured in saliva, or the inflammatory marker Interleukin-6 (IL-6), measured in blood, can also indicate the body’s stress level^5,6^.

A stressed state becomes “unhealthy” if the body is enforced to endure it over a longer period^7^. Dysregulation of the interaction between ANS and HPA axis, e.g., by failing to recover from the stressful situation, missing habituation to repeated stressors of the same type, or missing activation of one of the subsystems, can contribute to the negative impact of chronic stress^3,8^. There are various psychological and physiological consequences, such as cardiovascular diseases, inflammation, depression, and anxiety, that result from the dysregulation of the stress system^9^. Considering the severity of these consequences and the overall number of stress-related illnesses, we are in dire need of methods to manage this threat to our health, for instance, by developing interventions that prevent our stress system from deregulating.

Previous work has found that physical activity and mindfulness meditation^10^, yoga^11^, music listening^12^, tactile vibrations on the wrist^13^, HRV-based biofeedback^14^, or virtual reality, combined with olfactory interfaces^15^, have the potential to reduce psychological stress, mostly assessed by questionnaires. Rainforth et al.^16^ reviewed over 107 studies investigating the relationship between stress reduction and elevated blood pressure. Meta-analysis of 17 trials showed that stress intervention approaches like simple biofeedback, relaxation-assisted biofeedback, progressive muscle relaxation (PMR), and stress management training did not significantly reduce blood pressure as a response to stress. Only a transcendental meditation program resulted in statistically significant reductions.

Most works dealt with the reduction of either subjective stress measures or reactions of the SNS, such as heart rate and HRV. While cardiovascular activity is easy to assess, it is only an indirect measure of psychological stress since it can be influenced by various other factors. Only few approaches measured more direct determinants of acute stress, such as alpha-amylase for the SNS, or cortisol for the HPA axis. One example was presented by Heinrichs et al. who investigated the effect of oxytocin and social support on cortisol reactivity^17^. Thoma et al. showed that music listening before acute stress predominantly impacts the ANS (in terms of faster recovery of alpha-amylase levels after acute stress), and, to a lesser degree, the HPA axis and psychological factors^18^. Domes et al. reported that a 6-week training period of internet-based stress management (IBSM) and PMR both lead to lower subjective stress levels compared to a control group^19^. However, only IBSM was capable of significantly reducing the cortisol response to an acute stressor.

In summary, methods that biologically prevent the stress system from deregulating during *acute* stress, and, thus, might prevent the development of chronic stress in the first place, would be of great importance, but are still underexplored. One promising approach might be given by directly stimulating the PNS. Via links between the ANS and the HPA axis through the hypothalamus and the amygdala^3^, stimulating the PNS before an acute stressor may lead to a reduced stress response^20^, thus helping to recover better from a stressful situation. Electrical, transcutaneous stimulation of the vagus nerve, a major constituent of the PNS, has the potential to decrease sympathetic and increase parasympathetic function following stress exposure, as shown by Gurel et al.^21^.

Another, simpler approach for reliable vagal stimulation is presented by the diving response, a reflex present in all air-breathing vertebrates^22^ that is triggered by facial immersion in cold water. The excitation of the trigeminal nerve in the face, especially the ophthalmic and maxillary branch in the eye and forehead region, respectively, leads to stimulation of the vagus nerve through the trigeminal-vagal reflex arc^23^. Diving response-induced parasympathetic activation causes bradycardia and reduces blood flow to the limbs, while mean arterial pressure is slowly increasing^22^. A simplified and more unobtrusive way of triggering the diving response is given by the *Cold Face Test (CFT)* where a cold stimulus is applied to the face which also stimulates the trigeminal-vagal reflex arc in healthy individuals in a similar way as the diving response^24^. Khurana and Wu showed that bradycardia onset occurs, on average, 5.6 s after the beginning of the stimulus. Peak bradycardia was reached after about 35.8 s with a heart rate decrease of $22.5\% \pm 9.0\%$ (Mean ± SD)^25^.

Researchers have been using the CFT as a supportive measure in the diagnosis and research of neurological diseases affecting the autonomous nervous system^24^. It was reported that patients with diabetes mellitus, brainstem stroke, multiple sclerosis, or Shy-Drager syndrome showed less bradycardia—or even slight tachycardia—in response to the CFT^24^, compared to a control group. The CFT can not only aid in the diagnosis of diseases that directly affect the ANS but it can also be used to address psychosocial scenarios. Iorfino et al. conducted the CFT to investigate whether CFT-induced vagal excitation leads to increased prefrontal inhibitory control and, thus, causes individuals to perform better during a social cognition task. However, results contradicted the theory by showing that, even though the CFT increased high frequency (HF) HRV components, reflecting vagal tone, there was no improved social cognition performance^26^. La Marca et al. found associations between CFT-induced vagal activity and HPA axis reactivity. They showed that a faster heart rate response to the CFT is associated with reduced cortisol response to acute stress, suggesting an inverse relationship between vagal activity and HPA axis reactivity^27^. However, in all presented contributions the CFT was only used as means of diagnosis and not as an intervention. Using the CFT as an intervention method in everyday life appears to be feasible as it is non-invasive and easily applicable. Together with the advantage that active participation, such as breath-holding, is not required, the CFT has the potential of being a promising candidate for unobtrusive reduction of acute stress reactions.

For that reason, we examine whether systematically applying a cold facial stimulus might help us to fulfill our goal of increasing vagal activity during acute psychosocial stress, and. concurrently, inhibiting HPA axis activity. To the best of our knowledge, we are the first to apply the CFT as a direct intervention to reduce an acute stress response. With our work, we lay the foundation for further research in non-electrical vagal stimulation using cold facial stimulation.

## Methods

### Data acquisition

To assess whether CFT-induced vagal stimulation is capable of reducing an acute stress response we designed and conducted an experiment while concurrently recording electrophysiological and endocrinological features to capture the reaction of the human body to both acute stress and the CFT stimulus, respectively. In total, we recruited 28 young, healthy participants (82% female, age $20.1 \pm 2.5$ years, BMI $21.3 \pm 2.4\,\mathrm{kg\,m}^{-2}$) in psychology and engineering lectures at Friedrich-Alexander-Universität Erlangen-Nürnberg (FAU). The study was approved by the ethics committee of FAU (protocol #106_13B). All research was performed in accordance with relevant guidelines and regulations. Written informed consent was obtained from all participants before testing.

The study took place before the COVID-19 pandemic on several days between 11:00 a.m. and 5:30 p.m. Participants were asked to get up at least three hours before the study to minimize the impact of circadian variations in hormone concentrations^28^ and to avoid the consumption of alcohol on the day of the study and the preceding day. Participants were randomly assigned to either the Control condition or the condition that was exposed to the CFT. After arriving at the laboratory and completing the informed consent, a baseline saliva sample (*S0*) was collected to scan for (and possibly exclude) individuals with high cortisol baseline levels. Afterwards, participants were equipped with a wearable ECG sensor node (Portabiles GmbH, Erlangen, Germany) recording a 1-channel ECG according to Lead I of Einthoven’s triangle to assess ANS activity. After remaining rested on a chair for 15 min (denoted as *Global Baseline* or $BL_{Glo}$) they answered demographic and medical questionnaires to screen for possible exclusion criteria. The criteria were defined according to previous publications for acute stress induction (e.g., Janson and Rohleder^5^) and included: (1) a BMI lower than $18\,\mathrm{kg\,m}^{-2}$ or higher than $30\,\mathrm{kg\,m}^{-2}$, (2) the presence of physical or mental diseases, (3) medication intake (such as beta-blocker, glucocorticoids, anti-depressants, except contraceptives in women), (4) excessive consumption of alcohol ($>2$ alcohol beverages per day) or tobacco ($>5$ cigarettes per day), (5) previous experience with stress tests, and (6) self-reported depression, assessed by the “Allgemeine Depressionsskala” (ADS-L)^29^, the German version of the “Center for Epidemiological Studies Depression Scale” (CES-D)^30^. Participants that met any of the criteria were excluded and did not participate in any further steps of the study. Afterwards, the remaining participants were asked to fill out the “Multidimensionale Befindlichkeitsfragebogen” (MDBF), the German version of the “Mood State Questionnaire”^31^ to assess their mood state before the beginning of the stress task.

As stress protocol, we selected the Montreal Imaging Stress Task (MIST)^32^. While it was originally designed as a method to evaluate the impact of acute psychosocial stress on brain activation during functional magnetic resonance imaging (fMRI), a slightly modified version of the MIST protocol can be conducted as a computerized stress task while sitting at a desk. The protocol consists of a mental arithmetic challenge, implemented as a computer program with a graphical user interface, paired with a human investigator scrutinizing the performance of the individual as a social-evaluative threat.

Before beginning with the MIST, individuals were given time to practice the GUI for solving the arithmetic tasks (without time pressure and other evaluative elements) to get familiar with the interface. Afterwards, the MIST was conducted with both conditions. The MIST was divided into three phases (further denoted as MIST1-MIST3). Each MIST phase consisted of four subphases, beginning with the *Baseline* (BL) subphase (1 min), where no stressor was applied. Afterwards, the *Cold Face Intervention* (CFI) followed for the CFT condition and a *Resting Period* (RP) for the Control condition, respectively (2 min). The CFT was carried out by applying a cooling mask (Dr. Winkler GmbH, Ainring-Mitterfelden, Germany) to the face. The mask covered most facial areas with openings for the eyes, nose, and mouth. Therefore, normal breathing was ensured whilst triggering the oculocardiac reflex by applying pressure on the eyes was avoided^27^. The cooling mask was applied with a temperature of − 1 °C while participants sat upright on a chair and were instructed not to move or talk while continuing spontaneous breathing. An additional cooling mask (− 14 °C) was applied on top of the first one to prevent it from warming up too quickly.

The procedure was followed by the *Arithmetic Tasks* (AT) subphase (4 min). Participants were instructed to solve the arithmetic tasks displayed in the computer program and received immediate feedback on each answer. Besides the arithmetic tasks, the user interface contained various evaluative components, such as a performance evaluation bar that visualizes the user’s performance compared to a fictional average performance of other participants, constantly ranging between 80 and 90%, and a timeline indicating the remaining time for providing an answer. If a wrong (or no) answer had been given in the time interval, negative sound and feedback were provided. If the individual had answered three consecutive questions correctly the difficulty of the following task was increased. Contrarily, the difficulty was decreased if three successive incorrect answers were given.

Each MIST phase was concluded by the *Feedback* (FB) subphase (2–3 min), where the study instructor informed participants about their performances. During the first FB subphase, they were reminded that a minimum performance is required and that it must be close to the average performance in order not to be excluded from the study. The user interface was then explained again and participants were instructed to repeat the test. In the second FB interval, the study instructor entered the room and informed individuals about their repeated poor performance before consulting a (fictional) study leader about how to further proceed. After the study leader entered the room, participants were interrogated about personal problems (e.g., school performance) and were informed about the high costs and efforts of the study and the negative consequences if they had to be excluded due to insufficient performance. Finally, both the instructor and study leader remained in the room and asked participants to repeat the AT one last time under their direct supervision. The instructor, as well as the study leader, were of the same gender (male) for all participants. In total, the MIST protocol lasted about 30 min.

We chose the MIST over other protocols for acute psychosocial stress induction, such as the Trier Social Stress Test (TSST)^33^, because our intervention can be better integrated into the study protocol while concurrently allowing for more interventions. The MIST consists of three phases, thus, the CFT can be conducted three times immediately before each AT subphase. In contrast, the TSST would only allow two interventions: Before the public speaking and the mental arithmetic phase, respectively. Additionally, the MIST allows controlling better for other potentially disruptive factors such as postural changes, movement, or vocalization^27^ since the TSST is typically performed while standing in front of a panel of evaluators during the TSST.

**Figure 1:**
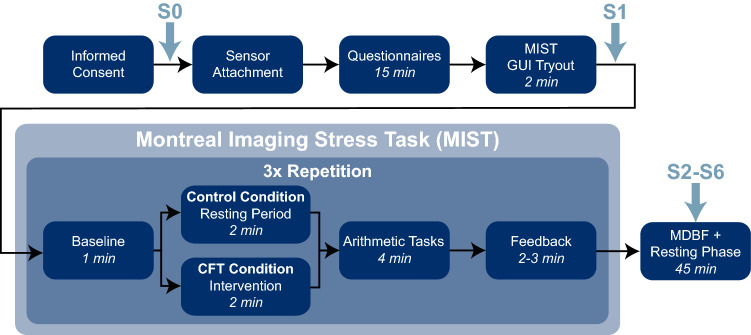
Overview of study protocol. The study consisted of a *Preparation Phase*, the *Montreal Imaging Stress Task (MIST*), and a *Resting Phase*. *S0–S6* indicate the time points at which saliva samples were taken.

Once the MIST was finished, participants were asked to answer the MDBF questionnaire once again to assess post-stress mood states and remained in the room for a concluding resting phase of 45 min. During that time, they were not allowed to use their smartphones but were instead asked to rest and/or read provided magazines. To assess HPA axis activity we collected a total of six saliva samples (S1–S6) along with the study procedure 0, 30, 40, 50, 60, 70 min relative to MIST start, respectively, using Salivettes (Sarstedt AG  & Co. KG, Nümbrecht, Germany). The participants were instructed to move the polystyrol swab around in the mouth in a circular motion for 2 min without chewing. Afterwards, the samples were stored at − 18 °C for later analysis in the laboratory. Finally, participants were debriefed and dismissed. The whole study procedure is also depicted in Fig. 1.

### Data processing

All data processing and statistical analysis were performed with *BioPsyKit*, our open-source Python package for the analysis of biopsychological data^34^. All (raw) data recorded during the experiment are available on Open Science Foundation (OSF) (https://osf.io/8fb6n/). The source code for data processing and for reproducing all analysis results, figures, and tables is available on GitHub^35^.

We used the ECG to compute heart rate (HR) and heart rate variability (HRV). For that, we recorded raw ECG sensor data at a sampling frequency of 256 Hz onto the internal storage of the sensor node. After the end of the study procedure, the raw data were transmitted to a computer as binary files for subsequent data processing. The signal was then first preprocessed by filtering with a second-order FIR bandpass filter (3–45 Hz) to reduce noise, such as powerline interference or baseline drifts. Subsequently, RR intervals were computed based on the R peaks extracted from the ECG signal after applying the QRS detection algorithm proposed by Hamilton^36^. We reduced RR interval artifacts by removing RR intervals corresponding to a heart rate $\le$ 45 bpm or $\ge$ 200 bpm, as well as by removing statistical outlier in RR intervals ($\ge 2.576\,\sigma$) and differences of successive RR intervals ($\ge 1.96\,\sigma$). Removed RR intervals were imputed by linear interpolation.

For computing HRV measures, R-peak locations were additionally corrected using an algorithm by Lipponen and Tarvainen^37^. Due to the fast cardiac recovery after the end of the cold face stimulus, only a short ECG interval is of interest to assess the CFT effect. Therefore, we selected the time-domain HRV measures *RMSSD* (root mean squared sum of successive RR interval differences) and *pRR50* (percentage of successive RR intervals differing by more than 50 ms) according to recommendations for assessing vagal tone^38,39^. We did not use the frequency-domain measure *HF* (high-frequency spectral power) to assess vagal tone since all of our intervals of interest were shorter than the recommended minimum duration of 5 min for frequency-based HRV analysis^38–40^. The HR(V) measures were computed over each individual subphase. We additionally normalized them with regard to $BL_{Glo}$ of each participant to reduce the effect of participant-dependent resting HR differences and to allow better comparison of results between individuals. Hence, all measures must be interpreted as percentage changes relative to $BL_{Glo}$, i.e., before any stress induction. To better assess the temporal effect of CFT-induced vagal stimulation we additionally computed the percentage of time one particular HR(V) measure was above the baseline value $BL_{Glo}$ during a MIST subphase (denoted as $\hat{t}_{Glo}$). For that, HR(V) measures were computed continuously using sliding windows with a window size of $N = 10$ samples. Thus, *higher* CFT-induced vagal stimulation, resulting in longer bradycardia, would lead to *lower* $\hat{t}_{Glo}(HR)$ values. In contrast, $\hat{t}_{Glo}(RMSSD)$ and $\hat{t}_{Glo}(pNN50)$ are expected to *increase*, respectively.

From the saliva samples, collected at time points $t_i$, we extracted raw cortisol concentrations ($c_i$). For that, we first centrifuged saliva samples at 2000*g* and 20 °C for 5 min. We then determined salivary cortisol concentrations in duplicate using a chemiluminescence immunoassay (CLIA, IBL, Hamburg, Germany) as described in more detail in previous publications (e.g., by Janson and Rohleder^5^).

The first saliva sample (S0) was disregarded from further analysis as it was only recorded for baseline comparison and potential exclusion of study participants. Apart from evaluating raw cortisol levels, we computed the area under the curve with respect to ground $AUC_G$ (Eq. 1) and the area under the curve with respect to increase $AUC_I$ (Eq. 2) over saliva samples S1–S6. $AUC_G$ serves as a measure for the total amount of cortisol secreted over time while $AUC_I$ is related to the sensitivity of the HPA axis to changes over time^41^. We additionally computed the maximum cortisol increase $\Delta c_{max}$ and the slope between S1 and S4 $a_{S1S4}$ as measures for the cortisol increase due to the stressor.

1 $$\begin{aligned} AUC_{G}= & {} \sum _{i=1}^{6} \frac{c_{i} + c_{i+1}}{2} \cdot (t_{i+1} - t_{i}) \end{aligned}$$

2 $$\begin{aligned} AUC_{I}= & {} AUC_{G} - c_{1} \cdot \sum _{i=1}^{6} \cdot (t_{i+1} - t_{i}) \end{aligned}$$

From the MDBF, recorded before and after the MIST, we computed the three dimensions “Good-Bad”, “Calmness-Nervousness”, and “Wakefulness-Tiredness” to assess the influence of the interplay between the stressor and the CFT intervention on different aspects of mood. Higher MDBF scores indicate better mood, higher calmness, and higher wakefulness, respectively.

### Statistics

For statistical analyses, all measures were first tested for normal distribution (Shapiro–Wilk test) and homogeneity of variances (Levene test). We then conducted two-tailed t-tests (or Mann–Whitney-U tests if the assumption of normal distribution was violated) to determine condition differences, mixed-measurement ANOVAs to determine possible interaction effects, as well as paired t-tests (or Wilcoxon signed-rank tests) and repeated-measurement ANOVAs to assess changes within conditions over time. Greenhouse-Geisser corrections were applied if the assumption of sphericity, indicated by the Mauchly Test, was violated. As post-hoc tests, we used pairwise t-tests and applied Bonferroni corrections within each HR(V) measure to counteract for multiple comparisons. *Condition* was used as a between-variable, whereas *Time*, *MIST phase*, and *MIST subphase*, respectively, were used as within-variables. The significance level $\alpha$ was set to 0.05. Effect sizes are reported as Hedges’ g for t-tests and $\eta _{p}^{2}$ with 95% confidence intervals for ANOVAs. In all Figures and Tables, we use the following notation to indicate statistical significance: *$p < 0.05$, **$p < 0.01$, ***$p < 0.001$.

## Results

### Participant exclusion

After data collection, three participants were excluded from further analysis: One participant did not respond to the CFT (even showing a slight tachycardia instead of the expected bradycardia) when no (*MIST1*) or only moderate stress (*MIST2*) was induced before. One participant showed a highly elevated initial (*S0*) cortisol level ($21.0\,\mathrm{nmol\,L}^{-1}$) that was higher than three standard deviations from the average S0 concentration of the study population and was thus considered a statistical outlier. Concurrently, another participant showed HR responses that were $>3\sigma$ from the average HR response. Thus, these participants were excluded as well. The remaining 25 participants (12 CFT, 13 Control) were included in the further analysis.

### Response to the MIST

**Figure 2:**
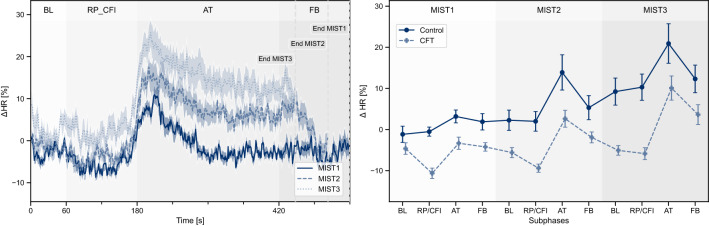
Left: Course of heart rate during the different *MIST* phases for both conditions combined, each consisting of *Baseline* (BL), *Resting Period / Cold Face Intervention* (RP/CFI), *Arithmetic Tasks* (AT), and *Feedback* (FB) subphases. Right: Average heart rate per MIST subphase during the conduction of the MIST. Values are depicted as mean and standard error.

To show that our study protocol reliably induced acute stress, we first investigated the responses of the Control condition to the MIST. During all MIST phases, the heart rate showed strong increases during the AT subphase, reaching peak tachycardia during the first minute of the AT interval (Fig. 2). Afterwards, the heart rate remained elevated for MIST2 and MIST3. During MIST1, maximum heart rate increases of $26.0\% \pm 10.5\%$ were observed which were even higher during MIST2 ($32.9\% \pm 15.4\%$) and MIST3 ($39.0\% \pm 17.1\%$). For each MIST phase, the heart rate increased significantly during AT compared to the preceding BL subphase while HRV measures decreased (Table 1), indicating an effective sympathetic activation due to the stressor.

Repeated-measures ANOVA of HR(V) measures during MIST subphases reveal increasing stress levels over time, expressed by a significant main effect MIST Phase during the subphases BL, FB, and AT (Table 2). Post-hoc testing showed significant HR differences between MIST1 and MIST3 for BL, $t(12) = -4.019, p = 0.005, g = -1.028$, and FB, $t(12) = -3.528, p = 0.013, g = -1.012$. During AT, HR differed significantly between MIST1 and MIST2, $t(12) = -2.832, p = 0.045, g = -0.892$, and between MIST1 and MIST3, $t(12) = -4.187, p = 0.004, g = -1.332$. We observed similar results for *RMSSD* and *pRR50* (Table S1). The increasing heart rate levels for the Control condition throughout the MIST protocol are also visible in Fig. 2 (right).

The stress-inducing effects of the MIST in the Control condition were further confirmed by self-reports. Mood, assessed by the MDBF questionnaire, decreased significantly in all MDBF dimensions as indicated by pairwise t-tests (Good-Bad: $t(12) = -5.801, p < 0.001, g = -1.904$, Awake-Tired: $t(12) = -2.985, p = 0.034, g = -0.617$, Calm-Nervous: $t(12) = -5.402, p < 0.001, g = -1.889$).

**Figure 3:**
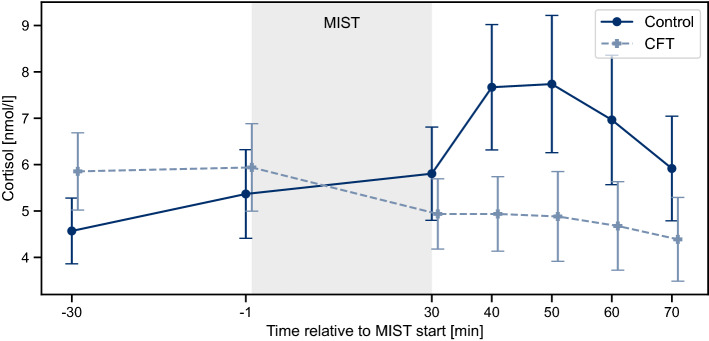
Cortisol response to the MIST of Control and CFT condition, respectively. Values are depicted as mean and standard error over all participants within one condition.

In addition, cortisol levels increased significantly after the MIST, $t(12) = 2.284, p = 0.041, g = 0.623$. Participants reached their peak levels between 10 min (*S3*) and 20 min (*S4*) after the end of the stress task and started to recover afterwards (Fig. 3).

**Table 1:** HR(V) responses to AT of the Control condition.

Measure	MIST1			MIST2			MIST3		
	*t*(12)	*p*	Hedges’ g	*t*(12)	*p*	Hedges’ g	*t*(12)	*p*	Hedges’ g
$\Delta HR$	3.311	0.019*	0.655	4.199	0.004**	0.894	3.850	0.007**	0.762
*RMSSD*	− 1.290	0.664	− 0.202	− 3.103	0.027*	− 0.534	− 1.936	0.230	− 0.351
*pRR50*	− 0.724	> 0.999	− 0.052	− 2.740	0.054	− 0.473	− 1.177	0.786	− 0.258

Paired t-tests were performed between BL and AT subphases for each individual MIST phase.

**Table 2:** HR(V) measures of the Control condition over the course of the MIST phases.

Measure	AT			BL			FB		
	*F*(2, 24)	*p*	$\eta ^2_p$	*F*(2, 24)	*p*	$\eta ^2_p$	*F*(2, 24)	*p*	$\eta ^2_p$
$\Delta HR$	9.160	0.001**	0.433	7.903	0.002**	0.397	9.249	0.001**	0.435
*RMSSD*	8.358	0.002**	0.411	2.466	0.106	0.171	7.398	0.003**	0.381
*pRR50*	8.189	0.002**	0.406	1.816	0.184	0.131	1.855	0.178	0.134

Repeated-measurement ANOVAs were performed separately for BL, AT, and FB subphases with MIST phase as within-variable.

### Response to the Cold Face Test

Applying the facial cooling mask resulted in CFT-induced vagal stimulation indicated by significant bradycardia during the CFI subphase for the first two MIST phases (Table 3). Peak bradycardia was $26.6\% \pm 18.8\%$ (MIST1), $23.9\% \pm 16.6\%$ (MIST2), and $20.1\% \pm 12.3\%$ (MIST3), compared to the preceding *BL* subphase, respectively. Concurrently, $\hat{t}_{Glo}(HR)$ during CFI was $9.39\% \pm 15.27\%$ (MIST1), $10.20\% \pm 8.98\%$ (MIST2), and $20.12\% \pm 17.51\%$ (MIST3), suggesting that, while the CFT was inducing parasympathetic activation in each iteration, the effect was decreasing with each MIST phase. *pRR*50 significantly increased during CFI in MIST1 and MIST3, respectively, while no significant differences were observed for *RMSSD* (Table 3).

**Table 3:** HR(V) responses of the CFT condition to the Cold Face Test.

Measure	MIST1			MIST2			MIST3		
	*t*(11)	*p*	Hedges’ g	*t*(11)	*p*	Hedges’ g	*t*(11)	*p*	Hedges’ g
$\Delta HR$	3.845	0.008**	1.255	2.972	0.038*	0.948	0.673	> 0.999	0.168
*RMSSD*	− 2.682	0.064	− 0.488	− 1.435	0.538	− 0.176	− 1.214	0.751	− 0.143
*pRR50*	− 3.149	0.028*	− 0.543	− 1.292	0.668	− 0.174	− 3.129	0.029*	− 0.415

Paired t-tests were performed between BL and RP/CFI subphases for each MIST phase separately.

### Effect of CFT on acute stress responses

As depicted in Fig. 2 (*right*) the average heart rate levels, compared to the global baseline $BL_{Glo}$ were lower for the CFT than for the Control condition throughout the whole study protocol. When comparing the relative HR increase between BL and AT, almost no response was found for the CFT condition during MIST1 while the Control condition showed a moderate response. However, during MIST2 and MIST3, both conditions reached similar high stress responses (Fig. 2, *right*, Table S2.1, and Table S2.2).

**Figure 4:**
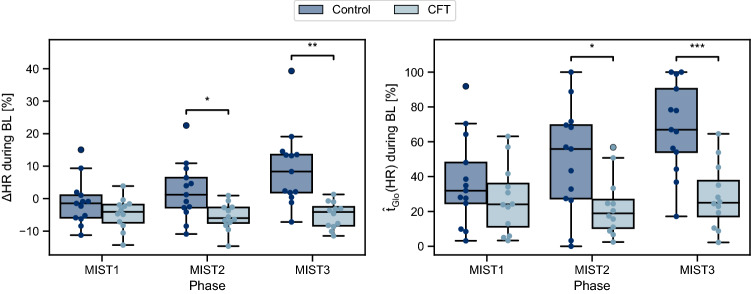
Differences in $\Delta HR$ (left) and $\hat{t}_{Glo}(HR)$ (right) between Control and CFT condition during BL for each MIST phase.

However, clear differences between both conditions can be observed in the BL subphases of MIST2 and MIST3, which were always performed before applying the CFT. Over the three MIST phases, the heart rate during BL remained roughly constant for the CFT condition, whereas it showed strong increases over time for the Control condition (Fig. 2, *right*, and Fig. 4). This drift of baseline levels in the Control condition is reflected by a significant interaction of *Condition* and *MIST Phase* in both *HR* and $\hat{t}_{Glo}(HR)$ (Table 4a). Post-hoc tests revealed that the heart rate during BL started to significantly differ in MIST2, $t(17.47) = -2.854, p = 0.032, g = -1.076$, and continued for MIST3, $t(14.98) = -4.092, p = 0.003, g = -1.533$. Similar results were observed for $\hat{t}_{Glo}(HR)$ (MIST2: $t(18.94) = -2.733, p = 0.040, g = -1.034$, MIST3: $t(21.70) = -4.420, p = 0.001, g = -1.688$) (Table S3). For the RP/CFI subphase, ANOVA revealed no significant interaction effects (Table S4), but significant main effects for *Condition* (Table 4b).

**Table 4:** Mixed-ANOVA results of HR(V) measures.

Measure	*F*(2, 46)	p	$\eta ^2_p$
(a) HR(V) measures during BL subphase (interaction effect MIST Phase $\times$ Condition)			
$\Delta HR$	6.261	0.004**	0.214
$\hat{t}_{Glo}(HR)$	3.473	0.039*	0.131
*RMSSD*	1.631	0.207	0.066
$\hat{t}_{Glo}(RMSSD)$	0.439	0.647	0.019
*pRR50*	1.315	0.278	0.054
$\hat{t}_{Glo}(pRR50)$	1.571	0.219	0.064

Measure	*F*(1, 23)	p	$\eta ^2_p$
(b) HR(V) measures during RP/CFI subphase (main effect Condition)			
$\Delta HR$	34.069	< 0.001***	0.597
$\hat{t}_{Glo}(HR)$	76.395	< 0.001***	0.769
*RMSSD*	0.052	0.821	0.002
$\hat{t}_{Glo}(RMSSD)$	14.443	< 0.001***	0.386
*pRR50*	0.382	0.543	0.016
$\hat{t}_{Glo}(pRR50)$	7.674	0.011*	0.250

Self-reported mood decreased slightly less for participants in the CFT condition compared to the Control condition, especially in the Awake-Tired dimension (Control: $11.85\% \pm 14.66\%$; CFT: $9.11\% \pm 20.24\%$). However, no significant interaction effects and main effects for condition were found (Table S5 and Table S6, Figure S1).

Cortisol responses of both conditions to the MIST are depicted in Fig. 3. ANOVA revealed a significant interaction of *Condition* by *Time*, $F(5,115) = 4.167, p = 0.002, \eta _p^2 = 0.153$. Additionally, participants in the CFT condition experienced significantly lower maximum cortisol responses compared to the Control condition, $U = 40.0, p = 0.041, g = -0.863$. Whereas the maximum cortisol increase was $71.49\% \pm 98.00\%$ for the Control condition, the CFT condition showed a maximum increase of $0.92\% \pm 32.37\%$. Similarly, $a_{S1S4}$ was significantly lower for the CFT condition, $U = 36.0, p = 0.024, g = -0.887$. $AUC_G$ and $AUC_I$ were both lower for the CFT condition, but the differences were not statistically significant (Table S7). Overall, cortisol measurements experienced high standard deviations.

## Discussion

The main objective of our study was to assess whether Cold Face Test (CFT)-induced parasympathetic stimulation before an acute stressor has the potential to reduce the following physiological stress response. Along with this goal, we investigated the inhibitory vagal effect on the HPA axis. The findings of our study reproduce those of prior applications of the MIST, such as by Dedovic et al.^32^ and La Marca et al.^27^: The MIST provokes a multidimensional stress response leading to vagal inhibition and HPA axis activation^27^. Successful HPA axis activation was confirmed by a significant increase in cortisol after the stressor. Each individual Arithmetic Task (AT) subphase elicited a statistically significant HR response compared to the preceding Baseline (BL). Moreover, HR constantly increased over the three AT subphases, suggesting an increase in stress load throughout the MIST. La Marca et al. reported that the MIST is a suitable protocol for effective stress-induced vagus nerve inhibition^27^. Accordingly, the HRV measures *RMSSD* and *pNN*50, both supposed to reflect vagal tone^38^, showed significant decreases. In addition, the MIST elicited not only physiological but also subjective stress responses. Self-reported mood, assessed by the MDBF, decreased significantly after the MIST in all three dimensions (Good-Bad, Awake-Tired, and Calm-Nervous) which was also reported by La Marca et al.^27^.

Applying the cold facial stimulus induced strong vagal activity shown by significant bradycardia during the CFI subphases in MIST1 and MIST2. The reached peak bradycardia levels are comparable with those of previous studies that involve healthy individuals, e.g., by Khurana et al.^24^, Reyners et al.^42^, Khurana and Wu^25^, and La Marca et al.^27^. Concurrently, HRV measures increased, supporting the assumption of successful parasympathetic stimulation even during phases of light stress and in the face of a recurring stressor. The effect of the CFT weakened with increasing stress exposure and did not induce significant bradycardia during MIST3. However, the heart rate during the third CFI was above the global baseline in only $20.12\% \pm 17.51\%$ of the time (assessed by $\hat{t}_{Glo}(HR)$). In total, we observed that the CFT seems to induce parasympathetic activity regardless of prior vagal inhibition and a possible anticipatory stress reaction in light of the next MIST phase. To the best of our knowledge, our study is the first to show this effect.

Nonetheless, the CFT condition showed a similar cardiovascular reaction to the MIST as the Control condition. Even though the heart rate responses to the stressor are smaller for the CFT condition during MIST1 and MIST2, compared to responses of the Control condition, participants of both conditions showed considerable HR increases, especially during MIST3 (Fig. 2, *right*). Hence, it appears that the CFT-induced parasympathetic stimulation right before the stress task was quickly abolished by the subsequently dominating MIST-induced vagal inhibition. However, considerable condition differences can be observed during the BL subphase. While the Control condition showed strong increases of $\Delta HR$ and $\hat{t}_{Glo}(HR)$ during BL over the three MIST phases both measures remained roughly constant for the CFT condition. This is supported by a significant interaction effect Condition $\times$ MIST Phase. The HRV measures *RMSSD* and *pRR*50 as well as $\hat{t}_{Glo}(RMSSD)$ and $\hat{t}_{Glo}(pRR50)$ followed this trend, although condition differences were not as distinct.

Remarkably, these differences occurred during all BL subphases, but not during the subphases AT and FB where acute psychosocial stress was induced. Since both conditions showed similar heart rate at the beginning of the first MIST phase, the continuously increasing heart rate levels of the Control condition during the three BL subphases indicate difficulties to return to the initial resting heart rate. In contrast, the CFT condition was mostly able to return to their pre-stress heart rate levels after each MIST phase (Fig. 2, right). While the underlying biological mechanism would need to be investigated further, it seems like the strong CFT-induced parasympathetic activation was only briefly suppressed during the AT subphase, but still present when the active stressor was removed. The preceding vagal stimulation appeared to help individuals to recover more efficiently between the MIST phases.

Stimulating the parasympathetic activity does not only affect physiological measures like HR(V), but also psychological measures like mood. Previous work has linked reduced HRV to negative mood^43,44^. Hence, limiting the stress-induced HRV decrease, or providing better recovery, might also limit mood worsening in this process. La Marca et al. have shown that applying the CFT leads to considerably less mood worsening after a stress task^27^. Our results, however, support these findings only partially. Although an effect of cold face stimulation, especially in the Awake-Tired dimension of the MDBF, might be assumed based on the findings of La Marca et al.^27^, condition differences were not significant. Hence, we cannot state that a CFT intervention influences mood worsening in a meaningful way. Nonetheless, we assume that promoting faster physiological recovery while concurrently reducing subjective stress perception could be even more promising for acute stress reduction. For that reason, future work should investigate this relationship in more detail.

In addition, our findings suggest that the CFT-induced vagal stimulation also seems to affect HPA axis activity. In comparison to the Control condition, the CFT condition showed a significantly decreased maximum cortisol response $\Delta c_{max}$ as well as a lower slope between S1 and S4 $a_{S1S4}$, and a statistically significant interaction Condition $\times$ Time. Furthermore, repeatedly applying the CFT during the MIST tends to decrease the total amount of secreted cortisol, indicated by a considerable decrease in the area under the cortisol curve $AUC_{G}$ for the CFT condition. We observed similar findings for the area under the cortisol curve with respect to increase $AUC_{I}$, a measure for HPA axis sensitivity^41^. Even though the condition differences were not significant, our results provide first indications that CFT-induced vagal stimulation has the potential to inhibit HPA axis activity during acute psychosocial stress. These findings are in line with theories suggesting a regulating effect of the vagus nerve on the HPA axis^45^, in particular the Polyvagal Theory^46^ or the Neurovisceral Integration model^47^, as well as with previous studies investigating associations between the parasympathetic nervous system and the HPA axis^27^. To investigate this effect further, future studies may help to ultimately make use of it in order to provide active intervention for helping to improve resilience to acute stress reactions.

However, the outcomes of our study might suffer from some limitations. Besides the relatively small number of participants included in the final analysis ($N=25$), the majority of study participants (82%) were female. It has been shown that the cortisol reactivity of females is influenced by the menstrual cycle and the use of oral contraceptives^48^, which was no exclusion criteria for this study. Furthermore, cortisol concentrations can generally be affected by several other biological factors such as, for instance, BMI and age^49^. Since participants were randomly assigned to one condition and both conditions were balanced with regard to sex, age, and BMI, biological factors should not have affected our results since they were present in both conditions. However, future work might need to investigate this in further detail and collect data from a study population that is better controlled for biological and medical influences. Furthermore, the study was conducted between 11:00 a.m. and 5:30 p.m.. As the curve of the diurnal cortisol concentrations reaches its flattest phase between 1:30 p.m. and 4:15 p.m. it is recommended to conduct stress studies only within this time slot to encounter diurnal cortisol fluctuations in the best possible way. However, the slope of the diurnal cortisol concentration decreases with the time after awakening^50^. To diminish the effect of decreasing cortisol levels during the stress task due to the diurnal rhythm, and to avoid influences of the cortisol awakening response, participants were asked to wake up at least three hours before the study. Additionally, we did not assess respiratory information during our experiment since our aim was to keep the number of sensors attached to the individuals as low as possible in order to keep potential distractions from the cold face intervention as little as possible. However, this leads to two drawbacks: On the one hand, this does not allow us to compute respiratory sinus arrhythmia which is supposed to reflect vagal tone^51^. On the other hand, we cannot assess the potential influence of the CFT on breathing and, thus, alterations in HR(V) due to changes in respiration patterns. Reyners et al. reported that continuing or stopping to breathe has an influence on the HR response to the CFT^42^. Thus, they recommend performing the CFT while not continuing to breathe in order to maximize the effect of the CFT. However, they did not report any indications on whether performing the CFT resulted in changes in respiration. Even though we instructed participants to continue regular and spontaneous breathing during the intervention, we can not fully rule out a potential confounding effect. For that reason, future studies should definitely consider assessing respiratory information and controlling the results for respiration.

## Conclusion and outlook

In this work, we explored the Cold Face Test (CFT) as an intervention method to interfere with acute stress reactions. For this purpose, we conducted the Montreal Imaging Stress Task (MIST) in a randomized between-subjects design. Our study confirmed the suitability of the CFT to partially stimulate vagal activity and inhibit HPA axis activity. We were able to show that CFT-induced bradycardia and, thus, increased parasympathetic activity, is present even in the light of mild and moderate stress exposure. Even though no differences can be observed during the actual stress task, the CFT condition recovered better from the stress situation, indicated by a significantly lower drift in baseline HR(V) levels over the MIST phases compared to the Control condition. We further found a significantly reduced cortisol increase after the MIST and a smaller area under the cortisol curve values for the CFT condition. Both findings count in favor of less secreted cortisol and decreased HPA axis reactivity.

Our work is the first to successfully use the physiological responses created by the CFT for enabling better recovery from acute stress. With our proof-of-concept study, we lay the groundwork for future work to better understand the underlying effects especially the interplay between SNS and PNS by investigating further HRV parameters, such as pre-ejection period (PEP), a cardiac marker for sympathetic activity^52^, or respiratory sinus arrhythmia as an index for parasympathetic activity^51^. These analyses could be complemented by assessing and analyzing potential condition differences in further, non-cardiac biomarker, such as alpha-amylase secretion (a non-cardiac marker for SNS activity^53^) or markers of immune system activation, such as Interleukin-6 (IL-6)^9^. Additionally, follow-up studies using a different stress protocol, such as the TSST, more participants, and better control for gender balance and biological factors influencing the stress response will help to generalize the findings of our current, initial study better.

## Supplementary Information

Supplementary Information.

## Data Availability

All (raw) data recorded during this study are available on OSF (https://osf.io/8fb6n/). The source code for data processing and for reproducing all analysis results and figures is available on GitHub (https://github.com/mad-lab-fau/cft-analysis).
