# Supplementary material for: Vagus activation by Cold Face Test reduces acute psychosocial stress responses
Source: Sci Rep. 2022 Nov 10;12:19270. doi: 10.1038/s41598-022-23222-9 (PMC9649023; doi:10.1038/s41598-022-23222-9)
Supplement: Supplementary file 1 — Supplementary Information. [file 41598_2022_23222_MOESM1_ESM.pdf]

Supplementary Information for Paper "Vagus Activation by Cold Face Test Reduces Acute Psychosocial Stress Responses"

R. Richer, J. Zenkner, A. Küderle, N. Rohleder, B. M. Eskofier

Supplementary Table S1

Posthoc results of HR(V) measures during the MIST. Pairwise t-tests were performed separately for BL, AT, and FB subphases between pairs of MIST phases.

			ΔHR			RMSSD			pRR50		
Subphase	*A*	*B*	$t(12)$	p	Hedges' g	$t(12)$	p	Hedges' g	$t(12)$	p	Hedges' g
BL	*MIST1*	*MIST2*	−1.378	0.580	−0.411	0.317	>0.999	0.050	0.527	>0.999	0.090
		MIST3	−4.019	0.005**	−1.028	1.861	0.262	0.439	1.768	0.307	0.377
	MIST2	*MIST3*	−2.399	0.101	−0.643	1.703	0.343	0.381	1.245	0.711	0.301
AT	*MIST1*	*MIST2*	−2.832	0.045*	−0.892	2.719	0.056	0.480	2.879	0.042*	0.532
		MIST3	−4.187	0.004**	−1.332	3.588	0.011*	0.658	3.388	0.016*	0.662
	MIST2	*MIST3*	−1.571	0.427	−0.414	1.397	0.563	0.204	0.934	>0.999	0.145
FB	*MIST1*	*MIST2*	−1.936	0.230	−0.367	0.847	>0.999	0.104	0.973	>0.999	0.130
		MIST3	−3.528	0.013*	−1.012	3.100	0.028*	0.514	1.442	0.525	0.300
	MIST2	*MIST3*	−2.755	0.053	−0.596	3.292	0.019*	0.383	1.480	0.494	0.171

Supplementary Table S2.1

Heart Rate increase between BL and AT subphases for each individual MIST phase. Values are depicted as M ± SD.

Condition / *Phase*	CFT	Control
MIST1	$1.3 \pm 5.6\,\%$	$4.34 \pm 4.73\,\%$
MIST2	$8.23 \pm 6.98\,\%$	$11.63 \pm 9.99\,\%$
MIST3	$15.16 \pm 8.09\,\%$	$11.69 \pm 10.95\,\%$

Supplementary Table S2.2

HR(V) responses to AT. Paired t-tests were performed between BL and AT subphases for each condition and each individual MIST phase, respectively.

				MIST1				MIST2				MIST3			
Condition	*Measure*	*A*	*B*	t	df	p	Hedges' g	t	df	p	Hedges' g	t	df	p	Hedges' g
CFT	ΔHR	*AT*	*BL*	0.803	11	>0.999	0.251	4.086	11	0.005**	1.359	6.491	11	<0.001***	1.886
	RMSSD	*AT*	*BL*	2.058	11	0.192	0.423	−1.404	11	0.564	−0.215	−3.911	11	0.007**	−0.461
	pRR50	*AT*	*BL*	2.315	11	0.123	0.475	−1.029	11	0.977	−0.216	−2.754	11	0.056	−0.370
Control	ΔHR	*AT*	*BL*	3.311	12	0.019*	0.655	4.199	12	0.004**	0.894	3.850	12	0.007**	0.762
	RMSSD	*AT*	*BL*	−1.290	12	0.664	−0.202	−3.103	12	0.027*	−0.534	−1.936	12	0.230	−0.351
	pRR50	*AT*	*BL*	−0.724	12	>0.999	−0.052	−2.740	12	0.054	−0.473	−1.177	12	0.786	−0.258

Supplementary Table S3

Post-hoc results (interaction effect MIST Phase x Condition) of HR(V) measures during BL subphase. Pairwise t-tests were performed for each MIST phase between Control and CFT condition.

	MIST1				MIST2				MIST3			
Measure	t	df	p	Hedges' g	t	df	p	Hedges' g	t	df	p	Hedges' g
ΔHR	−1.430	21.285	0.502	−0.545	−2.854	17.471	0.032*	−1.076	−4.092	14.976	0.003**	−1.533
$\hat{t}_{Glo}(HR)$	−1.098	22.179	0.853	−0.420	−2.733	18.937	0.040*	−1.034	−4.420	21.696	<0.001***	−1.688
RMSSD	−1.273	16.590	0.662	−0.479	−0.595	18.823	>0.999	−0.225	0.051	21.529	>0.999	0.020
$\hat{t}_{Glo}(RMSSD)$	0.269	23	>0.999	0.104	1.405	22.813	0.520	0.540	1.022	22.846	0.952	0.396
pRR50	−1.354	19.977	0.573	−0.514	−0.326	22.841	>0.999	−0.125	−0.294	20.864	>0.999	−0.112
$\hat{t}_{Glo}(pRR50)$	−0.356	22.823	>0.999	−0.138	1.044	21.031	0.925	0.408	1.453	22.898	0.479	0.562

Supplementary Table S4

Mixed-ANOVA results (interaction effect MIST Phase x Condition) of HR(V) measures during RP/CFI subphase.

Measure	$F(2,46)$	p	η_p^2
ΔHR	2.266	0.115	0.090
$\hat{t}_{Glo}(HR)$	3.369	0.043*	0.128
RMSSD	0.113	0.893	0.005
$\hat{t}_{Glo}(RMSSD)$	0.018	0.982	0.001
pRR50	0.034	0.967	0.002
$\hat{t}_{Glo}(pRR50)$	0.128	0.880	0.005

Supplementary Table S5

Mixed-ANOVA results (interaction effect Time x Condition) of self-reported mood, assessed via MDBF.

Dimension	$F(1,23)$	p	η_p^2
Good-Bad	0.009	0.923	0.000
Awake-Tired	0.087	0.770	0.004
Calm-Nervous	0.650	0.428	0.028

Supplementary Table S6

Mixed-ANOVA results (main effect Condition) of self-reported mood, assessed via MDBF.

Dimension	$F(1,23)$	p	η_p^2
Good-Bad	0.054	0.819	0.002
Awake-Tired	2.356	0.139	0.093
Calm-Nervous	0.176	0.678	0.008

Supplementary Figure S1

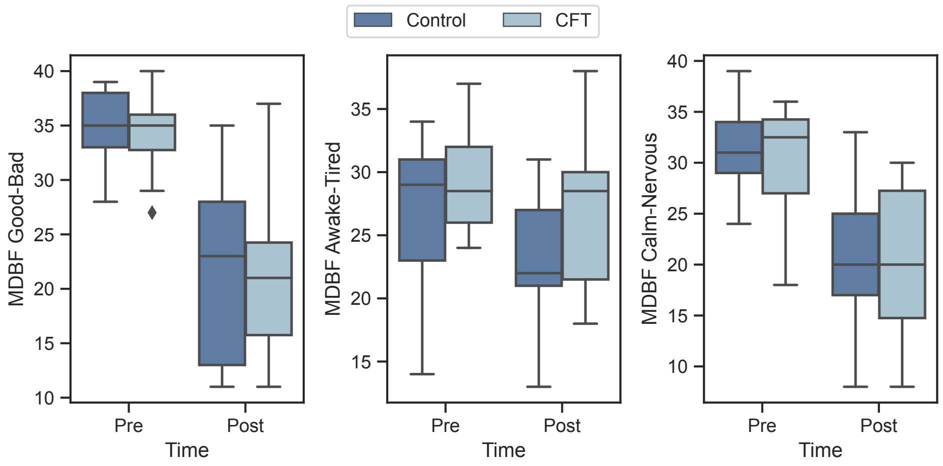

Self-reported mood, assessed via MDBF, before and after MIST for each condition individually.

Supplementary Table S7

T-tests of cortisol measures between Control and CFT condition.

Measure	U	p	Hedges' g
AUC_G	64	0.463	-0.380
AUC_I	44	0.068	-0.787
Δc_{max}	40	0.041*	-0.863
a_{S1S4}	36	0.024*	-0.887
